# ASSESSING MEDICAID PAYMENT RATES AND COSTS OF CARING FOR THE MEDICAID POPULATION RESIDING IN NURSING HOMES

**DOI:** 10.1093/geroni/igae098.2001

**Published:** 2024-12-31

**Authors:** Edward Miller, Elizabeth Simpson, Sara Karon

**Affiliations:** Chair; Co-Chair; Discussant

## Abstract

This symposium reports findings from an analysis of the relationship between Medicaid payment rates and costs for caring for the Medicaid population residing in nursing homes (NHs). Prior to the COVID-19 pandemic, there were about 1.37 million Americans using one of the nation’s 15,000+ NHs. Medicaid is the primary and largest single payer for NH care, covering approximately two-thirds of NH residents. The residents paid for by Medicaid’s nursing facility benefit are typically long-stay residents who often require custodial-type care due to cognitive or physical impairments that must be provided on a long-term basis. Until recently, there has been limited information examining the relationship between Medicaid payment rates and the costs of caring for Medicaid NH residents. Such information is essential to support discussion of policy reforms that aim to improve the safety and quality of NH care. This study used Medicaid payment and cost report data for freestanding NHs in 44 states collected during the latest pre-pandemic period (2019). The national average and median per diem Medicaid payment rates and costs and Medicaid payment-to-cost ratios were calculated for the nation as a whole and by NH characteristics and state Medicaid payment policies. The first presentation establishes the context for the study (Miller). The second presentation describes the study methodology (Simpson). The third presentation reports study findings (Cohen). The last presentation delineates implications of the study findings for policy and practice (Bowblis). Sara Karon will serve as discussant.

